# Multiple Phenotypic Changes Associated with Large-Scale Horizontal Gene Transfer

**DOI:** 10.1371/journal.pone.0102170

**Published:** 2014-07-21

**Authors:** Kevin Dougherty, Brian A. Smith, Autumn F. Moore, Shannon Maitland, Chris Fanger, Rachel Murillo, David A. Baltrus

**Affiliations:** School of Plant Sciences, University of Arizona, Tucson, Arizona, United States of America; University of Edinburgh, United Kingdom

## Abstract

Horizontal gene transfer often leads to phenotypic changes within recipient organisms independent of any immediate evolutionary benefits. While secondary phenotypic effects of horizontal transfer (i.e., changes in growth rates) have been demonstrated and studied across a variety of systems using relatively small plasmids and phage, little is known about the magnitude or number of such costs after the transfer of larger regions. Here we describe numerous phenotypic changes that occur after a large-scale horizontal transfer event (∼1 Mb megaplasmid) within *Pseudomonas stutzeri* including sensitization to various stresses as well as changes in bacterial behavior. These results highlight the power of horizontal transfer to shift pleiotropic relationships and cellular networks within bacterial genomes. They also provide an important context for how secondary effects of transfer can bias evolutionary trajectories and interactions between species. Lastly, these results and system provide a foundation to investigate evolutionary consequences in real time as newly acquired regions are ameliorated and integrated into new genomic contexts.

## Introduction

Horizontal Gene Transfer (HGT), the movement of genetic material between individuals without reproduction, is a major evolutionary force within microbial communities and impacts genome dynamics across all life [1], [2]. Although HGT events often provide direct fitness benefits to recipient cells, such as antibiotic resistance, integration of foreign DNA is an inefficient process [3]–[5]. As a result, newly acquired regions often interfere with physiological, genetic, and regulatory pathways to cause changes independent of phenotypes under immediate or direct selection pressures [6]. Numerous studies have demonstrated the existence of such costs by documenting changes to fitness, growth rate, or other phenotypes after the transfer of relatively small genomic regions. However, few studies have examined costs associated with megaplasmid transfer.

A variety of non-mutually exclusive mechanisms potentially contribute to costs of HGT. For instance, recently acquired genes are typically expressed at inefficient levels leading to limitations in resources such as ribonucleotides, amino acids, or ATP [7], [8]. Additional genes can occupy molecular machines required for basic cellular functions, such as polymerases and ribosomes, and sequester these limiting enzymatic resources from more critical activities [9], [10]. Foreign proteins may not fold correctly in their new cellular contexts, which could lead to disruption or triggering of stress responses [4], [11]. Recently acquired regions may disrupt flux through cellular systems, leading to the buildup of toxic intermediates [12], [13]. While such costs have been directly observed in laboratory experiments, retrospective studies across genomes add an additional layer of complexity as there exists an inverse correlation between gene retention after HGT and number of protein-protein interactions affected [14]. In most cases the precise molecular mechanisms underlying observed costs of HGT have not been identified, however, both the magnitude and molecular basis for costs could be greatly affected by both the size and gene content of the acquired region.

Costs of HGT have typically been studied by focusing on phenotypic changes after HGT of relatively small plasmids and lysogenic phage, even though large-scale transfers (>60,000 bp) occur at appreciable rates throughout bacteria [6], [15]–[17]. We have developed an experimental system to investigate the costs of large-scale HGT by using a ∼1Mb megaplasmid which is self-transmissible throughout *Pseudomonas* species [18]. Transfer of this megaplasmid occurs in both liquid and solid media and requires a type IV secretion system. This HGT event introduces approximately 700 ORFs into recipient cells, including many “housekeeping” genes as well as almost a full complement of tRNA loci as is characteristic of chromids [16], [19]. Importantly, it does not appear as though full pathways are present for megaplasmid encoded housekeeping gene pathways, so function very likely requires direct interaction with chromosomal networks.

In a parallel manuscript [18], we demonstrate that acquisition of this megaplasmid lowers fitness of *Pseudomonas stutzeri* by ∼20% and here we report on multiple additional phenotypes affected by large-scale HGT. Specifically, we find that megaplasmid acquisition leads to sensitivity to quinolone antibiotics, DNA intercalating agents, temperature, and killing by other bacterial species. We further find that HGT changes bacterial behavior in that biofilm formation is decreased and motility is increased. This widespread pleiotropy could signal that multiple phenotypic costs occur throughout transfer events [6]. Moreover, that such pleiotropic relationships between phenotypes are mediated at a systems level by single HGT events creates a unique situation where phenotypic evolution occurs as a by-product of evolutionary amelioration after transfer rather than direct selection on phenotypes themselves. In sum, we document the significant potential for secondary effects of HGT to alter phenotypic evolution and adaptive trajectories across microbial populations. This system further underscores the indirect power of costs of HGT to rapidly generate phenotypic diversity across closely related bacteria.

## Results

### Megaplasmid Acquisition Decreases Thermal Tolerance

**Figure 1:**
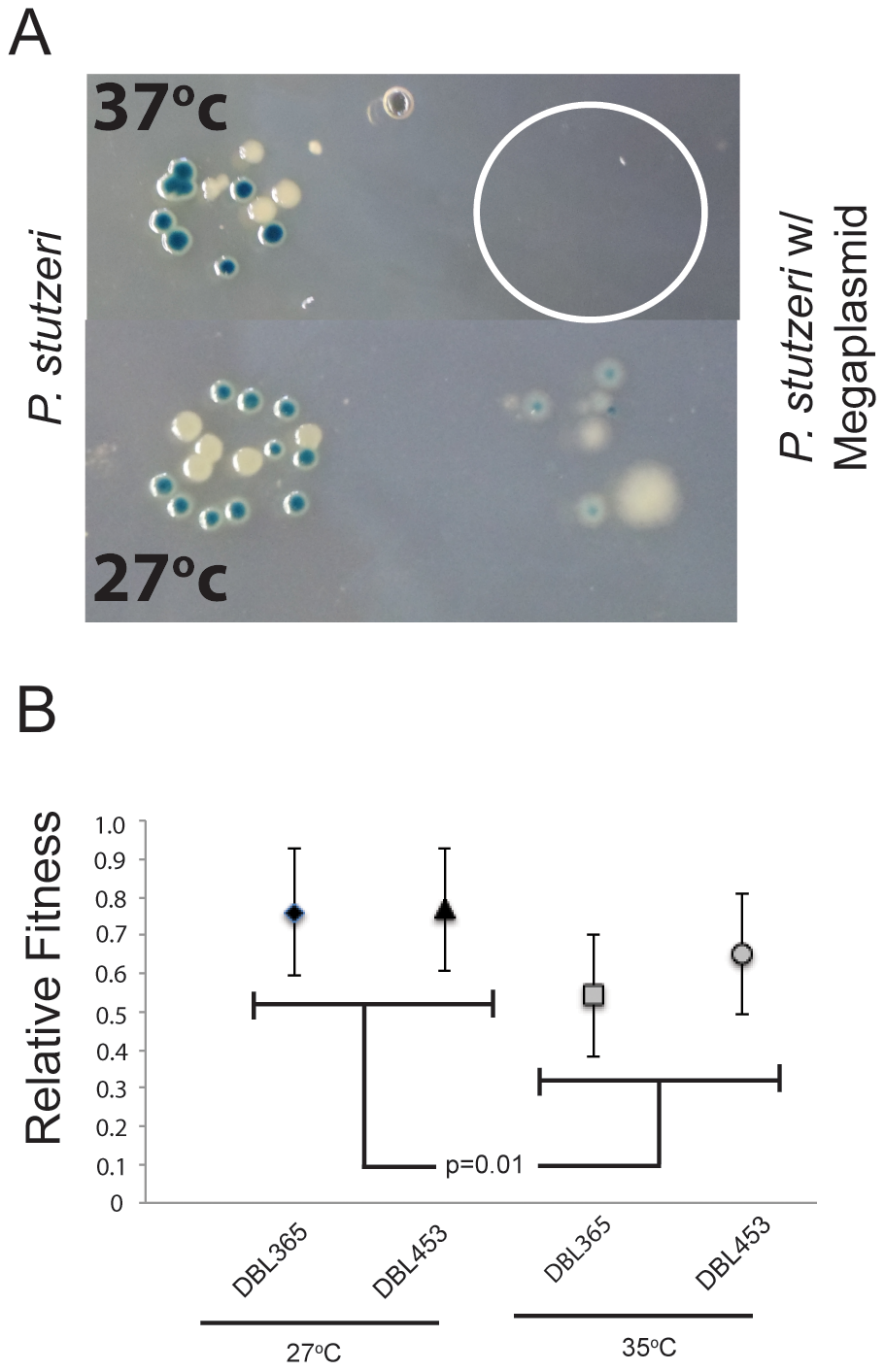
Megaplasmid Acquisition Decreases Thermal Tolerance. A) A dilution series of strains DBL332/DBL365 (white) and DBL390/DBL412 (blue), which lack/contain megaplasmid pMPPla107 were plated at either 27°C or 37°C. P. stutzeri normally grows at both temperatures (left), but strains containing the megaplasmid (right) only grow at 27oC. Pictures were taken at the lowest dilution with growth (approximately 1∶1×10^9^). White circle reflects where megaplasmid containing strains were spotted but didn't grow. B) Competitive fitness assays demonstrate that fitness of megaplasmid containing strains is significantly lower at 35°C compared to 27°C (p = 0.01). Fitness is normalized so that P. stutzeri lacking the megaplasmid is 1, error bars shown are +2 standard errors.

Our initial observations suggested that, although we were successfully able to select for conjugation of pMPPla107 from *P. syringae* pv. *lachrymans* 107 to *P. stutzeri* DBL332 when plated out on selective antibiotics at 27^o^c, conjugations failed when selected at 37^o^c (data not shown). We have since demonstrated, as one can see in figure 1A, megaplasmid acquisition sensitizes *P. stutzeri* to growth at 37°C and greater. Although both *P. stutzeri* DBL332 and DBL390 grow well at either temperature, independently created strains containing pMPPla107 (DBL365 and DAB412) appear stressed at 27°C and fail to grow at 37^o^c (Figure 1A). To further quantify this effect, we measured how changes in temperature alter competitive fitness for two related megaplasmid containing strains (DBL365 and DBL453). As one can see in figure 1B, presence of pMPPla107 decreases competitive fitness by 22% and 12% for DBL365 and DBL453 respectively at 35^o^c compared to 27^o^c. Analyzed within a full factorial ANOVA framework, this effect of temperature is significant (F_1,3_ = 45.33, p = 0.0067). Furthermore, analyzed as contrasts within the ANOVA framework, each strain's fitness is significantly lower at 35°C compared to 27°C (p<0.05). Since DBL453 is derived from DBL365 by recombining out the tetracycline marker, these results are not due to the marker itself. Therefore, two separate assays confirm that large-scale HGT of a megaplasmid decreases thermal tolerance in *P. stutzeri*.

### Megaplasmid Acquisition Decreases Antibiotic Resistance

**Figure 2:**
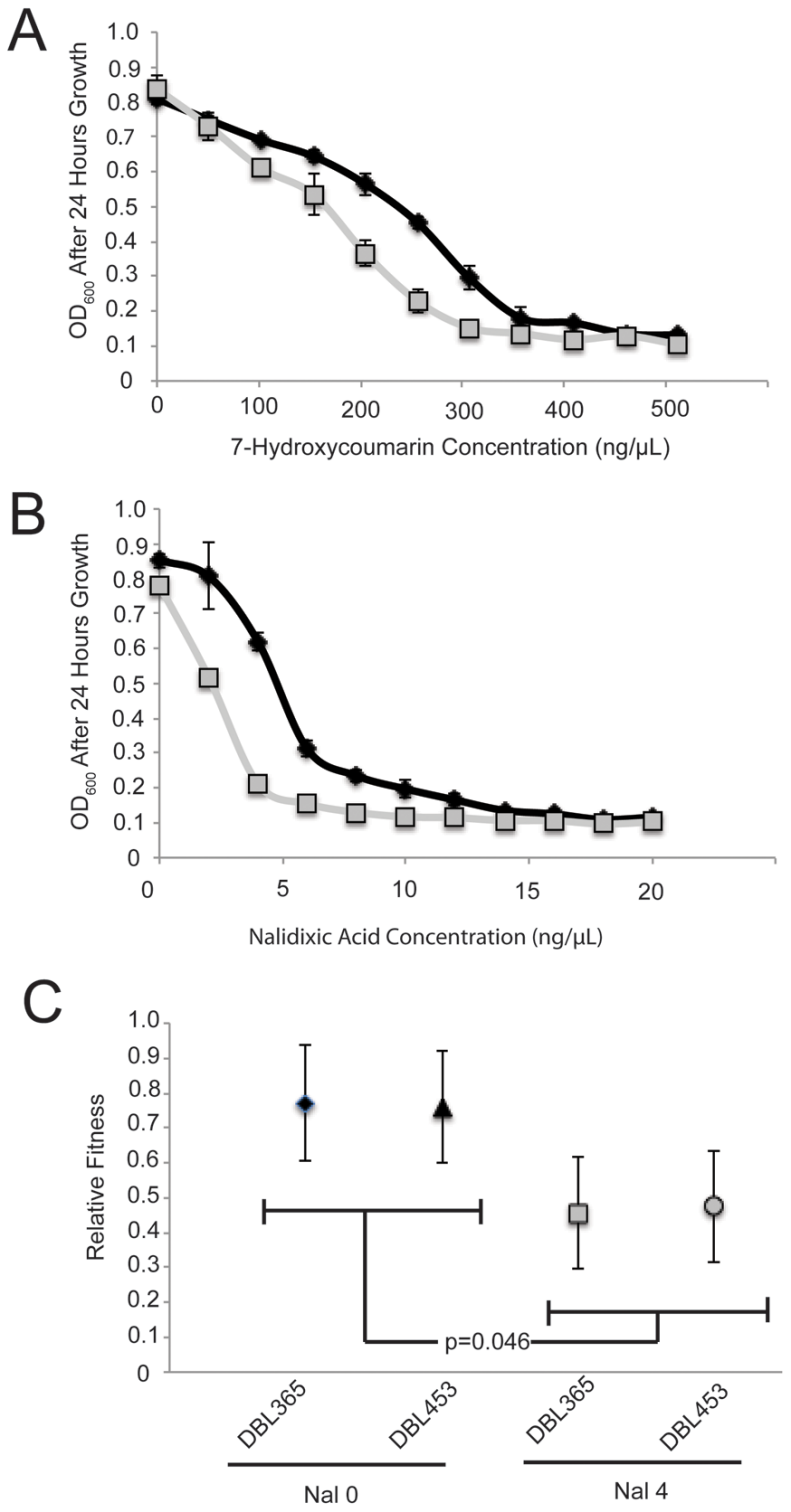
Megaplasmid Acquisition Decreases Resistance to 7-hydroxycoumarin and Nalidixic acid. A) A dose response curve comparing bacterial growth (OD_600_) of bacterial strains which contain (grey, DBL365) or lack (black, DBL332) the megaplasmid pMPPla107 across 7-hydroxycoumarin concentration. Curve is representative of at least 3 different assays. B) A dose response curve comparing bacterial growth (OD_600_) of bacterial strains which contain (grey, DBL365) or lack (black, DBL332) the megaplasmid pMPPla107 across nalidixic acid concentration. Curve is representative of at least 3 different assays. C) Competitive fitness assays demonstrate that fitness of megaplasmid containing strains is significantly lower in 4 µg/mL nalidixic acid compared to 0 µg/mL (p = 0.046). Fitness is normalized so that P. stutzeri lacking the megaplasmid is 1, error bars show +2 standard errors in all cases.

We used Biolog assays [20] to identify phenotypic changes associated with acquisition of megaplasmid pMPPla107 by *P. stutzeri* DBL187 (File S1). Overall, aside from an overall consistent negative effect of pMPPla107 on growth of *P. stutzeri,* acquisition of the megaplasmid only significantly changed a handful of phenotypes across replicates (File S1). One striking result is that megaplasmid acquisition decreases resistance of *P. stutzeri* to a variety of quinolone antibiotics as well as DNA intercalating agents such as 7-hydroxycoumarin. We followed up these results by performing replicated growth curves in both nalidixic acid and 7-hydroxycoumarin over a series of different concentrations after independently conjugating the megaplasmid into another *P. stutzeri* strain background (DBL332). As shown in figures 2A and B, megaplasmid presence lowers the minimal inhibitory concentration of *P. stutzeri* to both 7-hydroxycoumarin and nalidixic acid, thus replicating the results from the Biolog assay. To further quantify this difference in antibiotic resistance, we measured competitive fitness between DBL332 and DBL365 in 0 and 4 µg/mL nalidixic acid. As one can see in figure 2C, presence of pMPPla107 decreases competitive fitness by 31% and 28.5% for DBL365 and DBL453 respectively in the presence of 4 µg/mL nalidixic acid. Analyzed within a full factorial ANOVA framework, the effect of antibiotic is significant (F_1,2_ = 20.7564, p = 0.045). Furthermore, analyzed as contrasts within the ANOVA framework, each strain's fitness is significantly lower at 4 µg/mL nalidixic acid compared to standard SW-LB (p<0.05). Since DBL453 is derived from DBL365 by recombining out the tetracycline marker, these results are not due to the marker itself. Therefore, two separate assays confirm that large-scale HGT of a megaplasmid decreases resistance to quinolone antibiotics in *P. stutzeri*.

### Megaplasmid Acquisition Decreases Biofilm Formation

**Figure 3:**
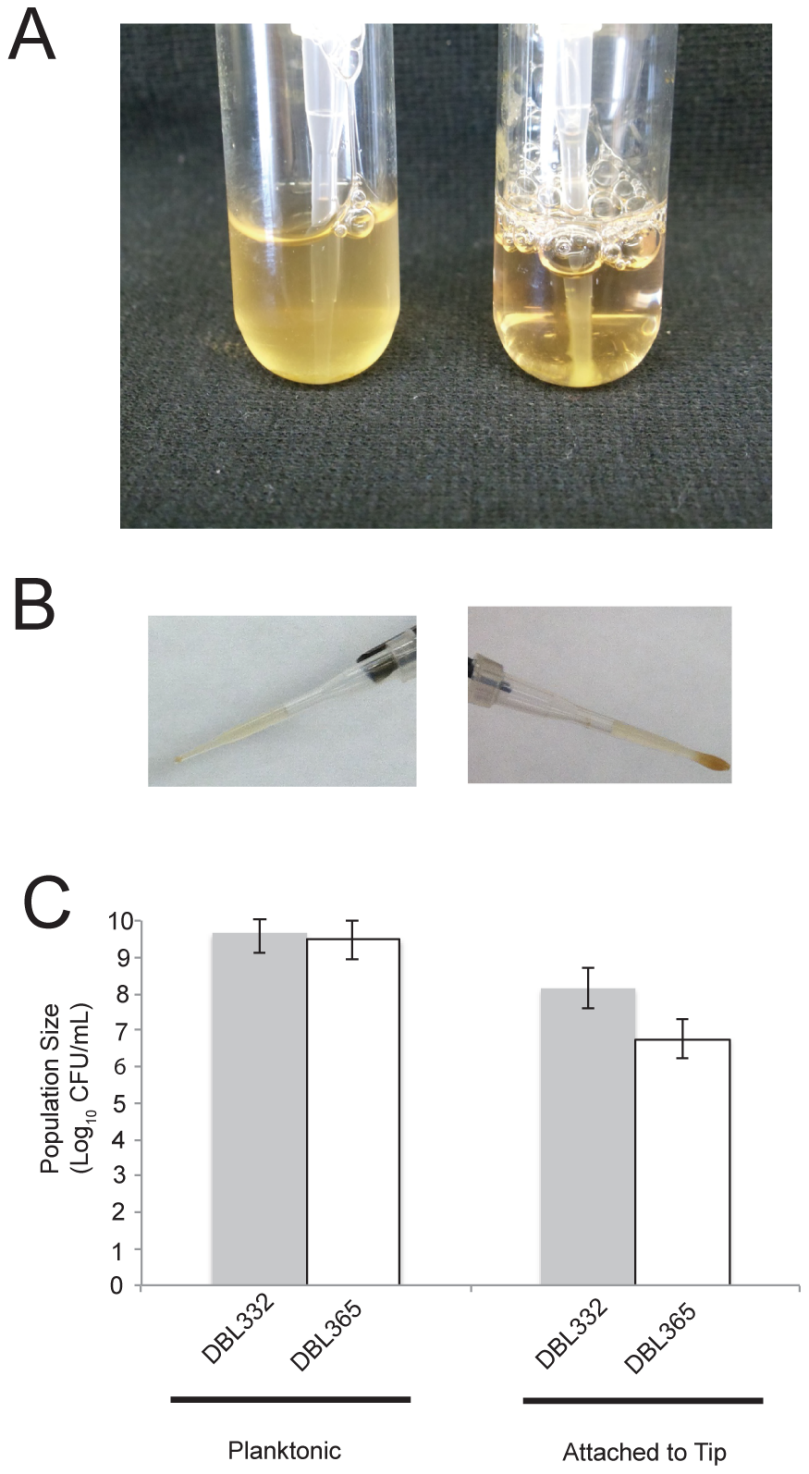
Megaplasmid Acquisition Decreases Biofilm Formation. A) Four day old cultures of strains which lack (DBL332, right) or contain (DBL365, left) megaplasmid pMPPla107 grown in M9 media supplemented with 10 Mm succinate. Total population sizes in both cultures are roughly equal, but turbidity differences arise because almost all cells without the megaplasmid are found within the biofilm mass. B) Pipette tips harvested after four days of growth in 2 mL SWLB media cultures for either DBL365 (left) and DBL332 (right). C) Bacterial population sizes of DBL332 (grey) and DBL365 (white) after four days of growth in 21mL cultures of SWLB media. Population sizes of planktonic bacteria are shown on the left, while those harvested from pipette tips are shown on the right. Error bars show +2 standard errors in all cases.

We witnessed that, after extended periods of growth in liquid media, *P. stutzeri* DBL332 forms a mass at the bottom of pipette tip within the culture after approximately 2-4 days (Figure 3A and B). This mass remains regardless of how long cultures are left to incubate (longest tested period was 10 days, data not shown). However, strain DBL365 does not form such a mass. To quantify this effect, we grew replicate cultures of strains DBL332 and DBL365 in SWLB media containing a single pipette tip. As one can see in figure 3C, even though population sizes of bacterial population suspended in liquid media are roughly equivalent (6.6×10^9^ and 4.8×10^9^ CFU/mL for DBL332 and DBL365 respectively), population sizes of bacteria attached to the pipette tip are much greater in the strain that lacks the megaplasmid (1.4×10^8^ compared to 7.6×10^6^). Therefore, whereas roughly 2% of *P. stutzeri* forms a mass on the pipette tip in the absence of pMPPla107, only 0.15% of the population forms this mass in a strain containing the megaplasmid. Similar results were observed for this assay with DBL453 as well as an additional *P. stutzeri* strain (DBL408) which acquired the megaplasmid independently of DBL365 (Figure S1). We also note that we attempted to perform traditional biofilm assays [21] to compare strains that contain or lack the megaplasmid, but slower growth of the megaplasmid strains made comparisons difficult to interpret (data not shown).

### Megaplasmid Acquisition Increases Motility

**Figure 4:**
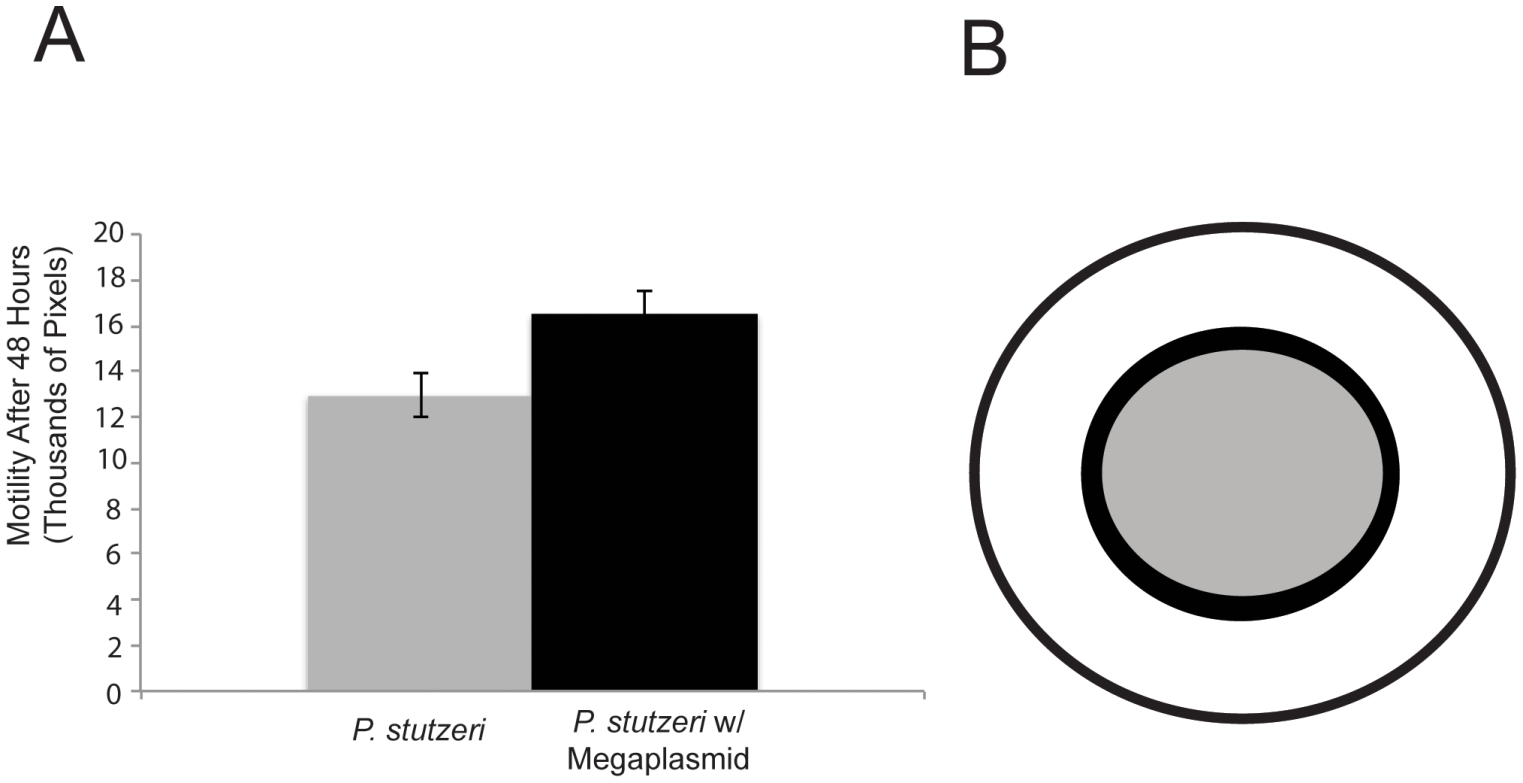
Megaplasmid Acquisition Increases Motility or Chemotaxis. A) Halo size in soft agar is significantly larger for bacterial strains which contain the megaplasmid (DBL365, black) compared to those that lack it (DBL332, grey). Error bars show +2 standard errors. B) Average difference in halo size represented in circular form. Grey circle is P. stutzeri while black circle represents P. stutzeri containing the megaplasmid.

We tested whether megaplasmid acquisition altered motility or chemotaxis in *P. stutzeri* using standard assays [22]. Briefly, 1 µL of liquid culture was plated into semisolid agar and strains were placed into an incubator. After two days, size of the bacterial halo surrounding the inoculation point was quantified. As shown in figure 4A, strains containing the megaplasmid consistently had larger halos than strains that lack the megaplasmid. Quantification shows that this increase in halo size is approximately 27% (F_1,4_ = 59.294, p = 0.0015). Similar results were observed for this assay with DBL453 as well as an additional *P. stutzeri* strain (DBL408) which acquired the megaplasmid independently of DBL365 (Figure S1).

### Megaplasmid Acquisition Increases Sensitivity to Supernatants from Other Bacterial Species

**Figure 5:**
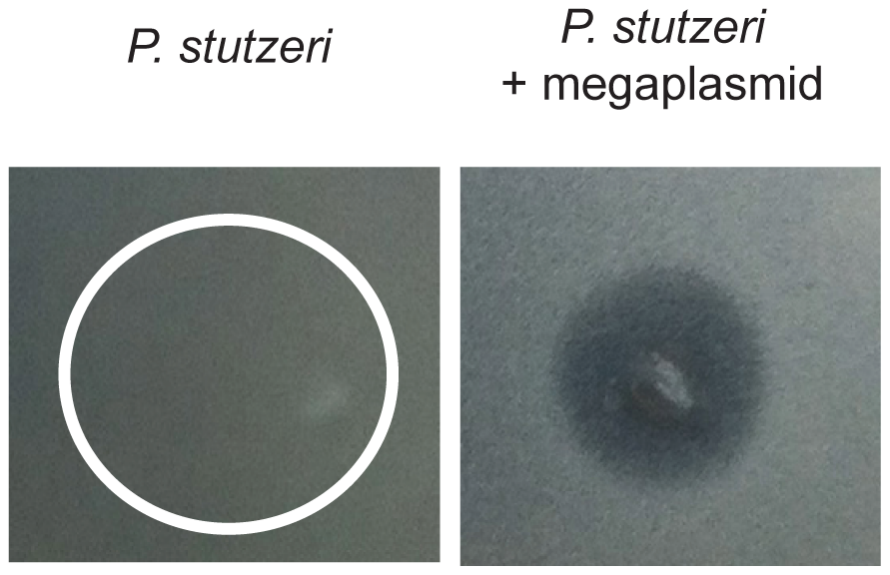
Megaplasmid Acquisition Increases Sensitivity to *P. aeruginosa* Supernatant. Purified supernatant from *P. aeruginosa* PA14 was spotted on lawns of *P. stutzeri* that either lacked (DBL332, left) or contained (DBL365, right) megaplasmid pMPPla107. White circle on left panel represents where supernatant was spotted onto lawn where *P.stutzeri* continued to grow. Right panel shows clearing area in lawns where supernatant was spotted. Photo is representative of >3 independent experiments.

*P. aeruginosa* strains are known to produce a variety of antimicrobial products during growth within laboratory culture, including pyocins and quinolones [23]–[25]. Since interactions between bacterial species occur frequently within the environment, and are essential for HGT of the megaplasmid to occur, we tested for whether megaplasmid acquisition altered sensitivity of *P. stutzeri* to *P. aeruginosa* supernatant. We found that supernatant from *P. aeruginosa* interfered with growth of *P. stutzeri* cultures, but only when strains contained the megaplasmid (Figure 5). Similar results were observed for this assay with an additional independently created *P. stutzeri* strain (DBL408, Figure S1). Therefore, megaplasmid acquisition alters interactions between bacterial species by sensitizing strains to growth inhibition by other bacterial species during normal growth.

## Discussion

For horizontally transferred regions to be maintained within a population, they must either provide a large enough benefit or be transmitted at high enough rates across individuals to avoid loss due to selection or genetic drift [26], [27]. That such benefits may be the primary target of strong selective pressures within a given environment, as with antibiotic resistance, doesn't preclude the existence of neutral secondary phenotypic changes or HGT-associated costs which are deleterious in other environments [6]. Although such costs of HGT appear to be widespread, there have been few efforts to investigate phenotypic effects of megaplasmid transfer. Furthermore, even when costs are observed, measurements are often limited to single phenotypes even though multiple cellular systems could be affected [5], [6], [13], [28]–[30]. Here we explore a system where HGT increases bacterial genome size by ∼20%, using a megaplasmid which is self-transmissible throughout pseudomonads [18], [19]. We report that megaplasmid acquisition alters numerous phenotypes within *P. stutzeri* in unprecedented ways, which highlights the potential for large-scale transfers to shape evolutionary dynamics within natural populations. This system provides a unique foundation to explore how evolution affects pleiotropic interactions between phenotypes altered as a result of large-scale HGT events, but also a powerful model to dissect individual interactions underlying these costs at a molecular level.

In a separate manuscript [18], we have demonstrated that megaplasmid acquisition by *P. stutzeri* impacts competitive fitness and bacterial growth under standard laboratory conditions. Here we show that megaplasmid acquisition is also accompanied by secondary changes to a variety of phenotypes affecting cellular physiology, environmental survival, and interactions with other species. For instance, megaplasmid acquisition increases sensitivity to quinolone antibiotics as well as stresses such as heat. To the best of our knowledge this is the first report of horizontal gene transfer directly lowering antibiotic resistance, and is especially interesting given that environmental stress can increase quinolone resistance [31]. That these responses are specifically affected by the test environments, as opposed to correlated effects on slower growth across a range of conditions, is highlighted by lack of sensitivity to a variety of other tested conditions (File S1). We also demonstrate that megaplasmid acquisition increases sensitivity to a substance present within the supernatant of *P. aeruginosa* cultures. As with quinolone sensitivity, to the best of our knowledge this is the first report of a plasmid mediating sensitivity to supernatant from bacterial cultures. Although we currently do not know the molecule(s) responsible for this effect, multiple bacteriocins and other antimicrobial targets known to be found within the supernatant will be the target of future studies [24], [25]. We have further shown that acquisition of the megaplasmid increases bacterial motility (or lowers the chemotaxis threshold) within soft agar and decreases biofilm formation in liquid culture. It is important to note that all of these phenotypic changes are effectively neutral under the defined laboratory conditions, which we use to select for successful conjugation, because this megaplasmid is engineered to provide tetracycline resistance. Therefore, under selective conditions in the lab, cells that can't acquire the megaplasmid will die due to antibiotic selection. While selective pressures in nature are likely more complex, these secondary changes could have dramatic effects on ecological strategies or niches between closely related bacterial populations.

This megaplasmid is representative of other large-scale gene transfers in terms of coding capacity, genetic content, and divergence from the recipient genome [15], [16], [19]. In spite of data demonstrating a negative bias for retention of highly connected genes after HGT events, megaplasmids often contain and can transfer numerous housekeeping genes [16]. The megaplasmid within our system itself contains 38 tRNA loci, polymerase subunits, DNA recombination and repair systems, a putative ribosomal protein, as well as other proteins that could be involved in housekeeping functions [19]. At the moment we don't know whether HGT associated costs are dependent upon any of these genes interfering with chromosomal pathways. However, results obtained herein could represent general outcomes after HGT events or may only be emergent properties of HGT by specific types of larger vectors like chromids.

One major question arising from these results concerns the independence of phenotypic shifts after HGT. Do all observed changes result from a single protein-protein interaction, numerous individual detrimental interactions, or the disruption of complex and interwoven regulatory networks? Furthermore, is the breadth of altered phenotypes a general property of HGT events as a whole or does the number of changes increase with size or gene content of transferred region? One candidate pathway does stand out as a potential mediator of these phenotypes *a priori*. Acquisition of the megaplasmid brings with it hundreds of new genes, the protein products of many of which are membrane localized [19]. Since cell size is limited, the incorporation of additional membrane bound proteins likely disrupts molecular signatures and dynamics of the membrane and could easily trigger or disrupt the envelope stress response [32]. The envelope stress response is conserved throughout bacteria, and responds to a variety of membrane stresses through the action of proteases, anti-sigma factors, as well as a host of other regulators. Since membrane integrity is critical for bacterial survival, the envelope stress response often sits at the top of regulatory cascades that control numerous phenotypes [33]. That previous results suggest quinolone sensitivity and sensitivity to *P. aeruginosa* supernatant are correlated with cell membrane integrity provides support for this model [34]–[36]. Furthermore, chaperone function links both heat and envelope stress responses, since one of the main determinants for both pathways is improperly folded proteins [32], [37], [38]. Lastly, motility and biofilm formation are directly regulated by the envelope stress response in *Pseudomonas*, as both the flagellum and pili are critical membrane bound structures [39], [40].

The addition of so extra DNA through HGT could also affect gene regulation at a global level through modification of chromosomal conformation. Proteins like HN-S play important roles in limiting detrimental effects of HGT by silencing transferred regions [41], [42], but are also critical for packaging the chromosome [43]. HN-S like proteins encoded by IncHI plasmids potentially mediate multiple phenotypic effects in after HGT in *Salmonella* including: increasing competitive fitness at low temperatures, increasing survival at high temperatures, and a reduction of motility [44]. MvaT is a *Pseudomonas* analog of HN-S, and an MvaT homolog (Pmr) found within the *Pseudomonas* pCAR1 plasmid mediates multiple phenotypic changes including increased resistance to chloramphenicol [13]. Plasmids often encode nucleiod associated proteins (NAPs) such as HN-S, so that regulatory alterations of chromosomally encoded pathways may be a fairly general feature of plasmid driven HGT [45]. It is striking that the phenotypes associated with acquisition of pMPPla107 by *P. stutzeri* are nearly the exact opposite of changes reported after HGT within for other systems. The pMPPla107 megaplasmid does contain at least three loci that resemble nucleoid associated proteins (including two divergent copies of MvaT/Pmr and a locus similar to IHF), but it is currently unknown whether interactions between these loci and chromosomal counterparts are responsible for observed costs. It is also possible that similar interactions between chromosome and plasmid occur across IncHI, pCAR1, and pMPPla107, but that *P. stutzeri* DBL332 regulates downstream pathways opposite of *Salmonella* and other pseudomonads.

Along these lines, quinolone antibiotics are known to disrupt gyrase function and alter the level of DNA supercoiling within the cell [36]. Sensitivity to quinolones could therefore arise after HGT because chromosomal maintenance requires a threshold amount of functional gyrase. Presence of the megaplasmid could increase this threshold or lower the amount of available gyrase, either of which would lower the concentration of quinolone required for antibiotic effects. Changes in DNA supercoiling are known to affect cellular responses to various stresses including heat shock [46]. However, one should note that a complex feedback loop exists between DNA supercoiling and the envelope stress response [47], and both may be important contributors to costs of HGT demonstrated within this manuscript. Alternatively, a variety of other independent physiological and regulatory pathways could be responsible for these changes including nutrient limitation triggering the stringent response or the disruption of multiple quorum sensing pathways altering regulation across the genome [48].

Outside of any direct fitness benefits from HGT, that multiple phenotypic changes are linked through single HGT events could skew evolutionary dynamics in unpredictable ways. For example, it is well known that adaptive trajectories and “evolvability” can be influenced by the order that beneficial or compensatory mutations fix [49], [50]. Secondary effects of HGT could bias future evolutionary paths within populations by altering magnitude or direction of epistatic interactions between adaptive mutations [51]. Furthermore, since recently acquired regions often function sub-optimally, the total number of potential beneficial mutations within an environment could be increased due to compensatory changes for the HGT event [52]. This influx of beneficial mutations correlated to HGT could impact both the order and magnitude of fixed adaptive mutations through clonal interference [53]. Along these lines, significant differences in evolutionary potential could arise based on, for instance, whether antibiotic resistance is introduced through HGT or *de novo* mutation. Such a balance could be further impacted in positive or negative ways by environment specific feedbacks on costs of HGT [54]. We also note that pMPPla107 conjugates across strains at high rates within the laboratory environment, on both solid media and in shaking liquid cultures, with transfer dependent on a type IV secretion system [18]. Therefore, the costs observed after HGT of this megaplasmid may not need to be compensated in order for pMPPla107 to be maintained within a population. These observations suggest an interesting line of research exploring interactions between costs of HGT, transmission rates, and megaplasmid persistence within populations through time.

Looking forward, amelioration of costs of HGT could lead to dramatic phenotypic differences between strains descended from a recent common ancestor due strictly to differences in paths of evolution and pleiotropic relationships from HGT [55]. These pleiotropic relationships could be reinforced or disrupted depending on which suites of mutations fix over time. For instance, amelioration of megaplasmid associated costs could produce one cluster of strains that is phenotypically indistinguishable from the non-megaplasmid ancestor while another cluster compensates only for costs at 27^o^c and is unable to grow at 37°C. Furthermore, as a result of costs of the megaplasmid to *P. stutzeri*, increased resistance to quinolone antibiotics such as nalidixic acid or ciprofloxacin could evolve solely due to compensation for the megaplasmid in the absence of direct selection by quinolones.

Since HGT introduces foreign genes and pathways into novel genomic contexts, each transfer event brings with it great potential to disrupt existing genetic and physiological networks within the recipient cell. Although numerous results have analyzed and dissected costs after the transfer of relatively small plasmids and phage, we have developed a model system with which to explore phenotypic costs associated with large-scale HGT (∼20%). The phenotypic shifts we see are dramatic and include changes of bacterial tolerance to numerous stresses as well as alterations of behavior.

Individually, a subset of these changes have been observed in other systems as a result of HGT or as a pleiotropic result of *de novo* adaptive mutations. However, taken as a whole, these results highlight the power of epistasis between the recipient genome and recently acquired regions to completely shift genetic and phenotypic expectations between closely related organisms. Furthermore, our results demonstrate the amazing breadth of phenotypes potentially affected by one HGT event and emphasize how singular evolutionary events can re-wire, reshape, and influence even the most well-studied genetic pathways.

## Materials and Methods

### Bacterial strains and plasmids

The ∼976 kb plasmid pMPPla107 was originally described in Pseudomonas syringae strain MAFF301305 (also known as pv. *lachrymans* 107) by Baltrus et al. 2011. A draft assembly sequence for pMPPla107 can be found at Genbank accession CM000959.1. All strains and plasmids used in the study are listed in Table 1. The focal *P. stutzeri* strain in this paper, 23a24, was isolated from soil [56], and was chosen for its high competence for natural transformation. This strain has been selected and phenotypically modified in a variety of ways to carry out the necessary experiments, with these modifications listed in Table 1.

**Table 1:** Strains and Plasmids.

Strain	Description	Phenotypes	Reference
DAB273	*Pseudomonas stutzeri* 23a24		[56]
DAB278	*Pseudomonas stutzeri* JM300		[58]
DAB282	A rifampicin resistant version of P. stutzeri JM300	RifR	This Paper
DBL332	A rifampicin resistant version of *P. stutzeri* 23a24	RifR	[18]
DBL365	pMPPla107 conjugated into DBL332 from DAB885	RifR, TetR, SucS	[18]
DAB386	Tn7 transposition from AKN84 into DBL332	RifR, KanR, StrepR, ChlorR, CFP+	[18]
DAB390	Tn7 transposition of pUC18-mini-Tn7T-Gm-lacZ into DBL332	RifR, GenR, LacZ+	[18]
DAB885	*P. syringae* pv. *Lachrymans* 107 with MTN1907 derivative in pMPPla107	RifR, TetR, SucS	[18]
DBL187	pMPPla107 conjugated into DAB282 from DAB885	RifR, TetR, SucS	This paper
DBL408	pMPPla107 conjugated into DAB388 from DAB885	RifR, TetR, KanR, StrepR, ChlorR, CFP+, SucS	This paper
DBL412	pMPPla107 conjugated into DAB390 from DAB885	RifR, GenR, LacZ+, TetR, SucS	This paper
DBL453	MTN1907 derivative recombined out of DAB365	TetR, RifR	This paper

Plasmid	Description	Antibiotics	Reference
pMPPla107	∼976 Kb Megaplasmid found naturally within *P. syringae* pv. *lachrymans*		[19]
MTN1907	Gateway destination vector for making clean deletions in *Pseudomonas*	TetR, KanR, CamR, SucS	[59]
pDONR207	Gateway entry vector from Invitrogen	GentR, CamR	Invitrogen
pDAB321	pDONR207 containing region from lac107	GentR	[18]
pDAB326	MTN1907 containing region from lac107	TetR, KanR, SucS	[18]
pUC18-mini-Tn7T-Gm-lacZ	Tn7 containing GenR, *lacZ*	GentR	[60]
AKN84	Tn7 containing KanR, ChlorR, StrepR, CFP+	KanR, CamR, StrepR	[61]

### Culture conditions

All experiments were carried out at 27°C unless otherwise stated. Salt water LB (SWLB) was used as base media for liquid cultures and agar plates [56]. All liquid cultures were incubated on a rotary shaker (200 rpm). Antibiotics were used at the following concentrations: 20 µg/mL tetracycline, 50 µg/mL kanamicin, 50 µg/mL rifampicin, 10 µg/mL streptomycin. Xgal was used at a concentration of 40 µg/mL. For competitive fitness experiments in the presence of nalidixic acid, a concentration of 4 µg/mL was used. All dilutions and cell suspensions took place in sterile 10 mM MgCl_2_.

### Competitive Fitness Assays

To quantify the fitness cost of pMPPla107 in multiple environments, we set up independent competitive fitness assays [57], for paired *P. stutzeri* strains (i.e., one that lacked (DBL332) & one that contained (DBL365) a megaplasmid tagged with tetracycline resistance) using a pMP-free control strain (DBL390) that was phenotypically marked with gentamycin resistance and *lacZ*. Each week, we revived the strains to LB agar plates and then SWLB liquid (containing tetracycline at 10 µg/mL for pMP strains) by inoculating overnight cultures with cells from the corresponding agar plate. Subsequently, because the pMP impairs growth (e.g., extends the length of lag phase), we normalized the growth phase among the strains by conditioning each strain in tet-free SWLB for 48 hours under competition conditions by inoculating 2 mL cultures with 5 µL of the revival culture and incubating at 27°C. Furthermore, because the cost of pMPPla107 is severe at elevated temperatures and in the presence of nalidixic acid, we skewed the initial ratio of the pMP strain relative to DBL390 (∼5∶1 test:control ratio), whereas the pMP-free strain was set up ∼1∶1 with DBL390. Briefly, using optical density (OD600) to normalize cell density, we made a competition master mix (MM) for each strain large enough to make eight replicate 2 mL competitions per environment (e.g., 60 mL for 8 replicate competitions in three environments). However, because Nal 4 µg/mL was one of the environments tested, we initially set up the master mix with 2X the cells per unit volume (e.g., cells for 60 mL in 30 mL) and independently diluted the master mix 50∶50 with Nal 8 (i.e., 10 mL MM & 10 mL Nal 8 SWLB). The remaining 20 mL of the MM was diluted with 20 mL Nal 0 and used for the 8-replicate 27C/Nal0 & 8-replicate 35C/Nal0 competitions. For each replicate competition, relative fitness was calculated as the ratio of the growth rate (i.e., number of doublings) of the strain tested (e.g., DBL332, DBL365) relative to that of DBL390 during direct competition. The number of doublings for each strain in each competition was calculated by comparing the final density (i.e., [CFU]) and ratio of each independent competition to the average initial estimate calculated from eight replicate serial dilutions of each MM.

### Thermal Tolerance Assays

Strains DBL332 and DBL365 were each grown up overnight in four replicate liquid cultures at 27°C in SWLB. A dilution series was then created from each tube and plated on two different SWLB agar plates. One plate was incubated at 27°C while the other was incubated at 37°C. This assay was carried out with two independently created megaplasmid strains (DBL365 and DBL412). After 4 days, both plates were photographed. Competitive fitness assays were performed as described above, with the following adjustments. The first set of replicate cultures was incubated for two days at 27°C, while the second set of replicates was incubated for two days at 35°C. 35°C was chosen as the temperature for competitions because DBL365 and DBL453 can undergo at least one doubling in these assays at this temperature. Each competitive fitness assay experiment contained 8 replicate cultures, and a total of 4 independent competitive fitness experiments were carried out for a total of 32 measurements for each strain. Within the ANOVA, strain and temperature were fixed variables while experiment was treated as a random variable.

### Antibiotic Resistance Assays

Strains DBL332 and DBL365 were each grown up overnight in liquid cultures at 27°C in SWLB. Each culture was then used to inoculate a 96-well plate containing varying concentrations of either nalidixic acid or 7-hydroxycoumarin. This 96-well plate was placed into a Biotek Synergy-H1 plate reader, and incubated at 27°C with shaking for 24 hours. Every hour we took measurements of OD_600_ from the assay plate in order to create a curve representing antibiotic inhibition. We chose 24 hours as the measurement represented in Figs. 1A and B because the starting OD_600_ values for each strain are equal. This assay was repeated with two independently created megaplasmid strains (DBL365 and DBL408), although only results from DBL365 are shown. Competitive fitness assays were performed as described above, with the following adjustments. A second set of replicates was inoculated into SWLB containing 4 µg/mL nalidixic acid. After two days, all cultures were plated out to SWLB agar plates containing Xgal, and the ratio of test/control strains was counted. Each competitive fitness assay experiment contained 8 replicate cultures, and a total of 3 independent competitive fitness experiments were carried out for a total of 24 measurements for each strain. Within the ANOVA, strain and antibiotic were fixed variables while experiment was treated as a random variable.

### Biofilm Assay

Strains DBL332 and DBL365 were each grown up overnight into 2 mL liquid cultures at 27°C in SWLB. After one day, a second culture was inoculated at a 1∶100 dilution in 2 mL from the initial overnight culture and a sterile micropipette tip (Rainin, 1–20 µL) was placed into the culture. This culture is incubated 4 days. At this point the pipette tip was extracted and thoroughly rinsed with 10 mM MgCl_2_ into a 2 mL Eppendorf tube containing 4 glass beads to remove biofilm from the tip. The tube was then shaken in a FastPrep machine for 20 seconds at 4 m/s. The dislodged bacteria were enumerated by plating a dilution series on SWLB, incubating at 27°C, and counting colonies after 3 days. Each assay experiment contained 4 replicate cultures, and a total of 4 independent competitive fitness experiments were carried out for a total of 16 measurements for each strain. Within the ANOVA, strain and population location were fixed variables while experiment was treated as a random variable.

### Motility Assay

Strains DBL332 and DBL365 were each grown up overnight in 2 mL liquid SWLB cultures at 27°C. Then, we normalized the cell density (OD_600_ of ∼1.0) for washed cell suspensions of each growth culture by suspending the harvested pellets in the appropriate volume of 10 mM MgCl_2_ (e.g., cells from I mL of OD_600_ = 2.0 were suspended in 2 mL). Using a sterile rounded wooden applicator (i.e, broken cotton tipped applicators), we inoculated the center of 2.5 mL motility agar (i.e., 1/2 strength LB and 0.25% agar) plates by first dipping the applicator into the washed cell suspensions. These plates were then incubated at 27°C for 48 hours, at which point pictures of each plate were scanned and the size of the motility halo was analyzed using ImageJ. Each assay experiment contained 8 replicate cultures, and a total of 5 independent motility assay experiments were carried out for a total of 40 measurements for each strain. Within the ANOVA, strain was a fixed variable while experiment was treated as a random variable.

### Bacteriocin Assay

*P. aeruginosa* strains with killing activity were grown overnight in LB liquid culture at 37°C with shaking. 2 mL were placed in a 2 mL Eppendorf tube and centrifuged at 10,000 rpm for 5 minutes. Supernatant was carefully filter sterilized into a fresh tube without disturbing the pellet. Target strains were grown overnight in LB at 27°C with shaking. A 1∶100 dilution in LB was made using the target strain overnight and grown for 4 hr. 300 µL were taken from these cultures and used to inoculate 3 mL of melted agar (0.4%). Inoculated agar tubes were mixed well and utilized as an overlay on desired medium plates. Let overlay solidify with plate lid on for about 10–20 min. Spot overlay with extracted killing supernatant and let dry. Incubate plates at 27°C for 1–2 days.

### Data

All raw data analyzed within this manuscript can be found within File S2.

## Supporting Information

Figure S1 **Phenotypic Changes Associated with Megaplasmid Acquisition by DBL408.** A) Competitive fitness assays demonstrate that fitness of DBL408 (containing pMPPla107) is significantly lower at 35°C compared to 27°C (p = 0.02). B) Pipette tips harvested after four days of growth in 2mL SWLB media cultures for either DBL332 (top) and DBL408 (bottom, contains pMPPla107). C) Halo size in soft agar is significantly larger for strains which contain the megaplasmid (DBL408, black) compared to those that lack it (DBL388, grey). D) Panel shows clearing area in lawns where supernatant was spotted onto an overlay with strain DBL408.
(TIF)

File S1 **Biolog Phenotype Assay Report for a Wild Type and Megaplasmid Containing Strain.** Biolog PM 1–20 were used to measure phenotypes within strains DAB282 and DBL187 (containing megaplasmid), with assays performed by Biolog. Two independent assays were run for each strain. This file is the word document received from Biolog that displays data from all assays highlights where there is a repeatable difference between strains over both assays and controlling for background growth effect of the megaplasmid.
(DOC)

File S2 **Relevant Raw Data from all Assays Reported Within this Study.** This data is contained within an Excel file, with data for each assay represented within different worksheets. Each worksheet (except antibiotic MIC curves) contains columns denoting the strain used, numerical experimental result, treatment, and experimental block. Each worksheet also contains a picture of the statistical analysis of these treatments. For raw data within competitions, we have not included adjusted raw fitness compared to the wild type strains competed at same time but these did not affect statistical treatments. For antibiotic MIC curves, we include optical density data for four replicate assays after 24 hours of growth for each strain measured within the same 96 well plate. These data are reported over a range of antibiotic concentrations.
(XLSX)
