# Supplementary figures and images for: Multiple Phenotypic Changes Associated with Large-Scale Horizontal Gene Transfer
Source: PLoS One. 2014 Jul 21;9(7):e102170. doi: 10.1371/journal.pone.0102170 (PMC4105467; doi:10.1371/journal.pone.0102170)

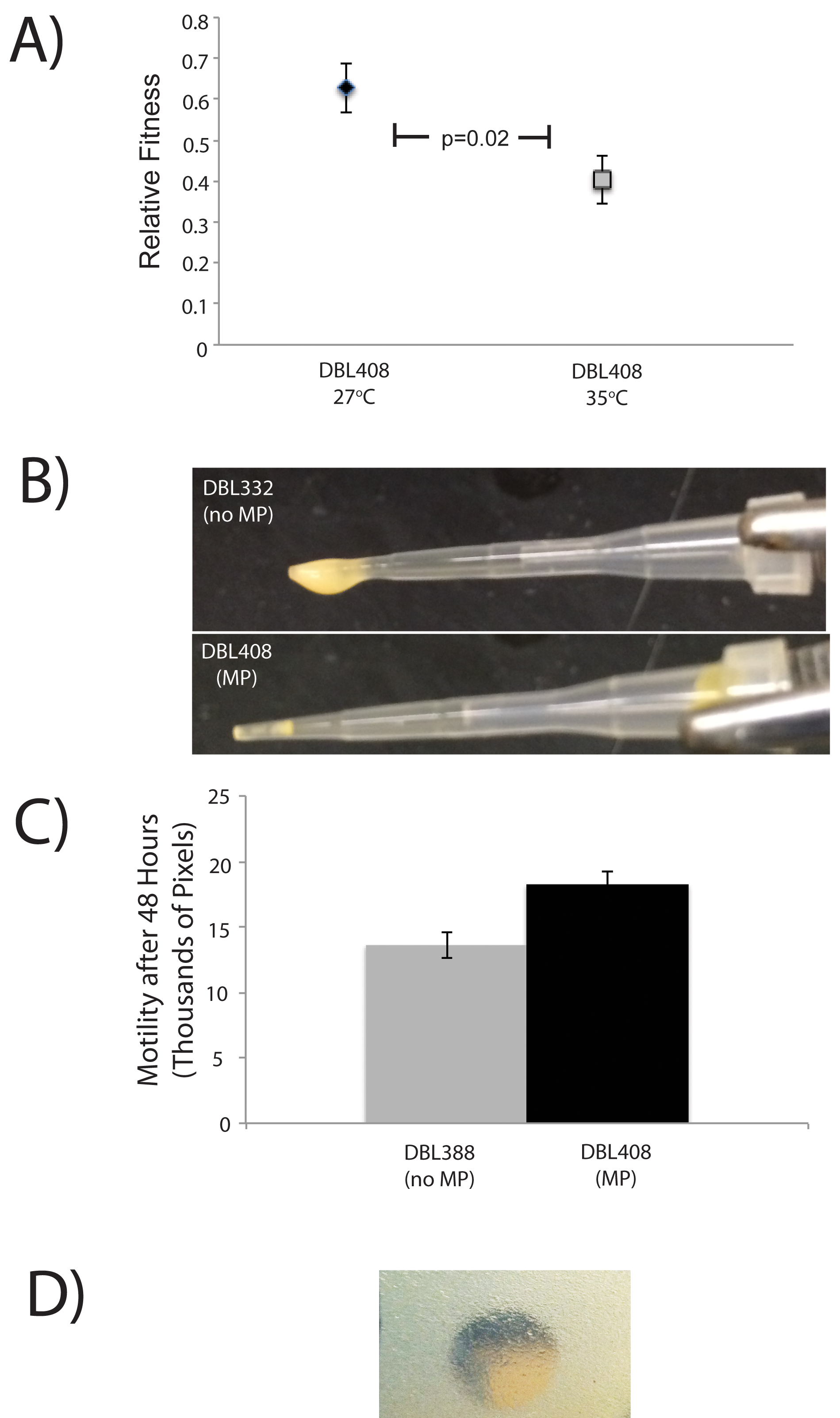

Supplement: Figure S1 — Phenotypic Changes Associated with Megaplasmid Acquisition by DBL408. A) Competitive fitness assays demonstrate that fitness of DBL408 (containing pMPPla107) is significantly lower at 35°C compared to 27°C (p = 0.02). B) Pipette tips harvested after four days of growth in 2mL SWLB media cultures for either DBL332 (top) and DBL408 (bottom, contains pMPPla107). C) Halo size in soft agar is significantly larger for strains which contain the megaplasmid (DBL408, black) compared to those that lack it (DBL388, grey). D) Panel shows clearing area in lawns where supernatant was spotted onto an overlay with strain DBL408. (TIF) [file pone.0102170.s001.tif]
