# Supplementary material for: Multiple Phenotypic Changes Associated with Large-Scale Horizontal Gene Transfer
Source: PLoS One. 2014 Jul 21;9(7):e102170. doi: 10.1371/journal.pone.0102170 (PMC4105467; doi:10.1371/journal.pone.0102170)
Supplement: File S1 — Biolog Phenotype Assay Report for a Wild Type and Megaplasmid Containing Strain. Biolog PM 1–20 were used to measure phenotypes within strains DAB282 and DBL187 (containing megaplasmid), with assays performed by Biolog. Two independent assays were run for each strain. This file is the word document received from Biolog that displays data from all assays highlights where there is a repeatable difference between strains over both assays and controlling for background growth effect of the megaplasmid. (DOC) [file pone.0102170.s002.doc]

PstuSIDx6277xDBL187 versus PstuSIDx6276xDAB282

BiOLOG

21124 Cabot Blvd.

Hayward, CA 94545

Phenotype MicroArray Analysis

Test: Pstu_SID_6277_DBL187

Reference: Pstu_SID_6276_DAB282

Report Date: 4/24/2012 2:53:58 PM

Reproducibiltiy Analysis:

Test Reference

PM01: 00: passes PM01: 00: passes

PM02: 00: passes PM02: 00: passes

PM03: 00: passes PM03: 01: passes

PM04: 03: passes PM04: 03: passes

PM05: 01: passes PM05: 00: passes

PM06: 00: passes PM06: 00: passes

PM07: 00: passes PM07: 03: passes

PM08: 02: passes PM08: 00: passes

PM09: 06: passes PM09: 01: passes

PM10: 11: passes PM10: 00: passes

PM11: 05: passes PM11: 02: passes

PM12: 02: passes PM12: 05: passes

PM13: 05: passes PM13: 01: passes

PM14: 01: passes PM14: 00: passes

PM15: 06: passes PM15: 06: passes

PM16: 03: passes PM16: 02: passes

PM17: 03: passes PM17: 01: passes

PM18: 04: passes PM18: 02: passes

PM19: 10: passes PM19: 03: passes

PM20: 06: passes PM20: 01: passes

PM21: -1: N/A PM21: -1: N/A

PM22: -1: N/A PM22: -1: N/A

PM23: -1: N/A PM23: -1: N/A

PM24: -1: N/A PM24: -1: N/A

PM25: -1: N/A PM25: -1: N/A

PM26: -1: N/A PM26: -1: N/A

PM27: -1: N/A PM27: -1: N/A

PM28: -1: N/A PM28: -1: N/A

PM29: -1: N/A PM29: -1: N/A

PM30: -1: N/A PM30: -1: N/A

PM31: -1: N/A PM31: -1: N/A

PM32: -1: N/A PM32: -1: N/A

PM33: -1: N/A PM33: -1: N/A

PM34: -1: N/A PM34: -1: N/A

PM35: -1: N/A PM35: -1: N/A

PM36: -1: N/A PM36: -1: N/A

PM37: -1: N/A PM37: -1: N/A

PM38: -1: N/A PM38: -1: N/A

PM39: -1: N/A PM39: -1: N/A

PM40: -1: N/A PM40: -1: N/A

Replicate 1 versus Replicate 2 of Test:

Pstu_SID_6277_DBL187


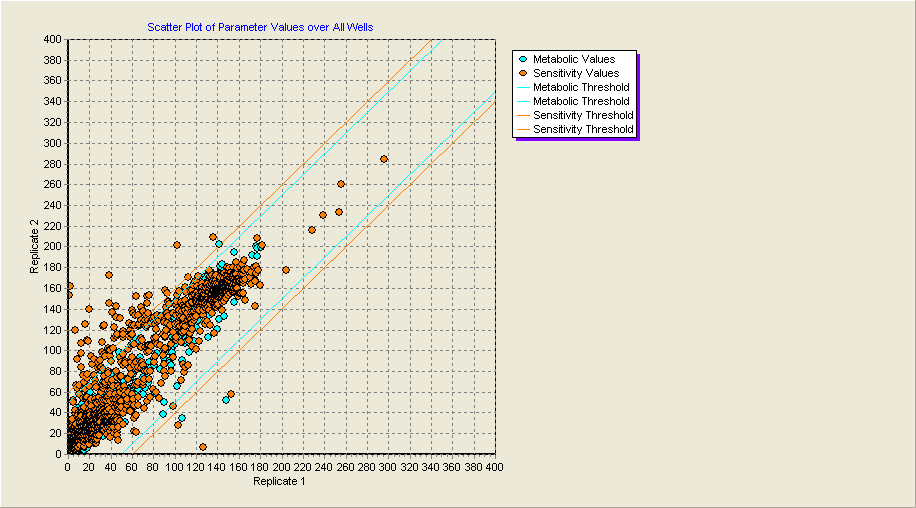


Replicate 1 versus Replicate 2 of Reference:

Pstu_SID_6276_DAB282


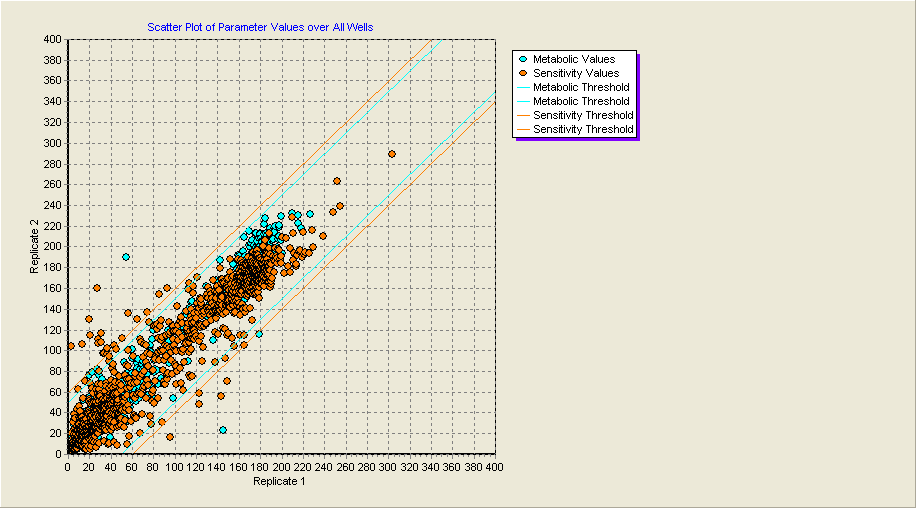


Run 1:

Pstu_SID_6277_DBL187( green )

versus

Pstu_SID_6276_DAB282( red )


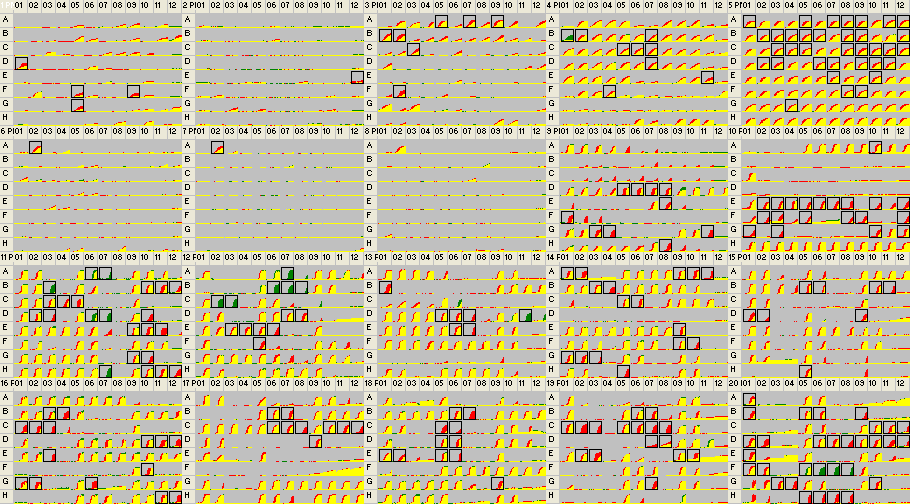


Run 2:

Pstu_SID_6277_DBL187( green )

versus

Pstu_SID_6276_DAB282( red )


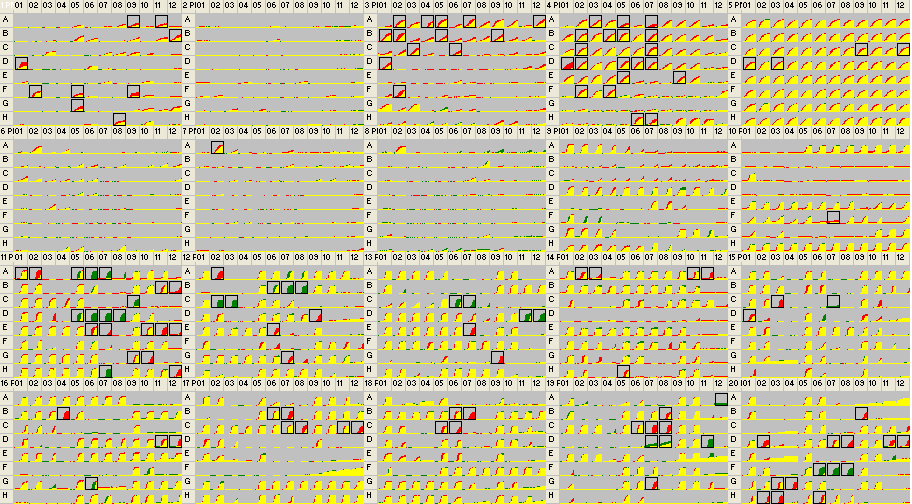


Consensus:

Pstu_SID_6277_DBL187( green )

versus

Pstu_SID_6276_DAB282( red )


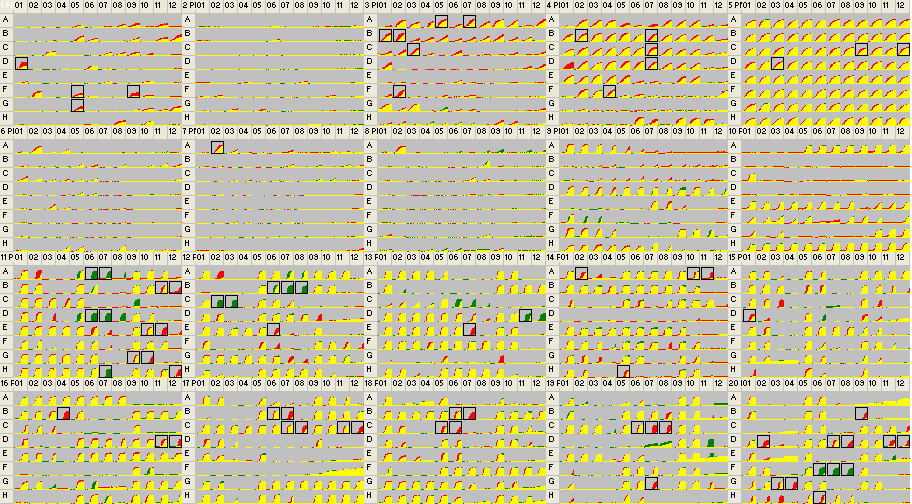


PM Report:

Pstu_SID_6277_DBL187_

versus

Pstu_SID_6276_DAB282_

Phenotypes Gained:

PM12B C02,C03 238 Paromomycin protein synthesis, 30S ribosomal subunit, aminoglycoside

PM11C H07 128 Kanamycin protein synthesis, 30S ribosomal subunit, aminoglycoside

PM20B F06,F07,F08 378 Oxytetracycline protein synthesis, 30S ribosomal subunit, tetracycline

PM11C D06,D07 299 Demeclocycline protein synthesis, 30S ribosomal subunit, tetracycline

PM12B B06,B07,B08 288 Penimepicycline protein synthesis, 30S ribosomal subunit, tetracycline

PM11C A06,A07 182 Chlortetracycline protein synthesis, 30S ribosomal subunit, tetracycline

PM13B D11 117 Rolitetracycline protein synthesis, 30S ribosomal subunit, tetracycline

Phenotypes Lost:

PM14A A10,A11 -108 Sanguinarine chloride ATPase, Na+/K+ and Mg++

PM01 D01 -106 L-Asparagine C-Source, amino acid

PM01 G05 -56 L-Alanine C-Source, amino acid

PM01 F09 -89 Glycolic acid C-Source, carboxylic acid

PM01 F05 -55 Fumaric acid C-Source, carboxylic acid

PM15B D01 -67 Phleomycin DNA damage, oxidation

PM19 C06,C07,C08 -228 7-Hydroxycoumarin DNA intercalator

PM20B D02 -101 Proflavine DNA intercalator, inhibits RNA synthesis

PM14A A02 -74 Acriflavine DNA intercalator, inhibits RNA synthesis

PM18C B06,B07 -159 Pipemidic Acid DNA topoisomerase

PM11C E10,E11 -156 Nalidixic acid DNA topoisomerase

PM11C B11,B12 -148 Lomefloxacin DNA topoisomerase

PM20B D07,D08 -140 Ciprofloxacin DNA topoisomerase

PM16A D11,D12 -114 Cinoxacin DNA topoisomerase

PM16A B04 -113 Norfloxacin DNA topoisomerase

PM11C H12 -90 Ofloxacin DNA topoisomerase

PM17A C11,C12 -113 Sulfamonomethoxine folate antagonist

PM18C C05,C06 -123 Sulfisoxazole folate antagonist, PABA analog

PM17A C07,C08 -118 Sulfachloropyridazine folate antagonist, PABA analog

PM12B E06 -73 Sulfadiazine folate antagonist, PABA analog

PM20B G03,G04 -114 Captan fungicide, carbamate

PM20B H06 -70 Tolylfluanid fungicide, phenylsulphamide

PM14A H05 -65 Promethazine membrane, phenothiazine, efflux pump inhibitor, anti-psychotic

PM03B B02 -88 Glycine N-Source, amino acid

PM03B A07 -70 L-Alanine N-Source, amino acid

PM03B B01 -65 L-Glutamine N-Source, amino acid

PM03B C03 -64 D-Alanine N-Source, amino acid

PM07 A02 -59 L-Glutamine N-Source, amino acid

PM03B F02 -85 Adenine N-Source, other

PM03B A05 -73 Urea N-Source, other

PM05 C09 -55 (5) 4-Amino-Imidazole-4(5)-Carboxamide Nutritional supplement

PM05 C12 -54 2`-Deoxyinosine Nutritional supplement

PM05 D03 -50 Chorismic acid Nutritional supplement

PM04A B02 -53 Dithiophosphate P-Source, inorganic

PM04A C07 -64 6-Phospho-Gluconic acid P-Source, organic

PM04A B07 -59 D-3-Phospho-Glyceric acid P-Source, organic

PM04A D07 -53 O-Phospho-L-Threonine P-Source, organic

PM17A B06,B07 -129 Hygromycin B protein synthesis, 30S ribosomal subunit, aminoglycoside

PM13B E07 -88 Geneticin disulfate (G418) protein synthesis, 30S ribosomal subunit, aminoglycoside

PM19 G07 -79 Dihydrostreptomycin protein synthesis, 30S ribosomal subunit, aminoglycoside

PM20B D11,D12 -128 18-Crown-6 ether respiration, ionophore, H+

PM20B B09 -63 Tetrazolium violet respiration, uncoupler

PM04A F04 -53 Tetrathionate S-Source, inorganic

PM11C G09,G10 -105 Potassium tellurite toxic anion

Appendix:

Carbon and Nutrient Max Read Hour: 048

Nitrogen, Phosphorus, Sulfur Max Read Hour: 048

Sensitivity Max Read Hour: 048

Metabolic Distance Threshold: 050

Sensitivity Distance Threshold: 060

Test Data Files:

Replicate 1:

PM01 : J:\Service\Customer\300\348\data\assay\9379\pstu_SIDx6277xDBL187___1_27_379_125_Aprx11x2012_A_15A.oka

Replicate 2:

PM01 : J:\Service\Customer\300\348\data\assay\9391\pstu_SIDx6277xDBL187___1_27_391_114_Aprx16x2012_B_11A.oka

Replicate 1:

PM02A: J:\Service\Customer\300\348\data\assay\9379\pstu_SIDx6277xDBL187___2_27_379_125_Aprx11x2012_A_15B.oka

Replicate 2:

PM02A: J:\Service\Customer\300\348\data\assay\9391\pstu_SIDx6277xDBL187___2_27_391_114_Aprx16x2012_B_11B.oka

Replicate 1:

PM03B: J:\Service\Customer\300\348\data\assay\9391\pstu_SIDx6277xDBL187___3_27_391_114_Aprx16x2012_B_12A.oka

Replicate 2:

PM03B: J:\Service\Customer\300\348\data\assay\9379\pstu_SIDx6277xDBL187___3_27_379_125_Aprx11x2012_A_16A.oka

Replicate 1:

PM04A: J:\Service\Customer\300\348\data\assay\9391\pstu_SIDx6277xDBL187___4_27_391_114_Aprx16x2012_B_12B.oka

Replicate 2:

PM04A: J:\Service\Customer\300\348\data\assay\9379\pstu_SIDx6277xDBL187___4_27_379_125_Aprx11x2012_A_16B.oka

Replicate 1:

PM05 : J:\Service\Customer\300\348\data\assay\9391\pstu_SIDx6277xDBL187___5_27_391_114_Aprx16x2012_B_13A.oka

Replicate 2:

PM05 : J:\Service\Customer\300\348\data\assay\9379\pstu_SIDx6277xDBL187___5_27_379_125_Aprx11x2012_A_17A.oka

Replicate 1:

PM06 : J:\Service\Customer\300\348\data\assay\9391\pstu_SIDx6277xDBL187___6_27_391_114_Aprx16x2012_B_13B.oka

Replicate 2:

PM06 : J:\Service\Customer\300\348\data\assay\9379\pstu_SIDx6277xDBL187___6_27_379_125_Aprx11x2012_A_17B.oka

Replicate 1:

PM07 : J:\Service\Customer\300\348\data\assay\9391\pstu_SIDx6277xDBL187___7_27_391_114_Aprx16x2012_B_14A.oka

Replicate 2:

PM07 : J:\Service\Customer\300\348\data\assay\9379\pstu_SIDx6277xDBL187___7_27_379_125_Aprx11x2012_A_18A.oka

Replicate 1:

PM08 : J:\Service\Customer\300\348\data\assay\9391\pstu_SIDx6277xDBL187___8_27_391_114_Aprx16x2012_B_14B.oka

Replicate 2:

PM08 : J:\Service\Customer\300\348\data\assay\9379\pstu_SIDx6277xDBL187___8_27_379_125_Aprx11x2012_A_18B.oka

Replicate 1:

PM09 : J:\Service\Customer\300\348\data\assay\9379\pstu_SIDx6277xDBL187___9_27_379_125_Aprx11x2012_A_19A.oka

Replicate 2:

PM09 : J:\Service\Customer\300\348\data\assay\9391\pstu_SIDx6277xDBL187___9_27_391_114_Aprx16x2012_B_15A.oka

Replicate 1:

PM10 : J:\Service\Customer\300\348\data\assay\9379\pstu_SIDx6277xDBL187___10_27_379_125_Aprx11x2012_A_19B.oka

Replicate 2:

PM10 : J:\Service\Customer\300\348\data\assay\9391\pstu_SIDx6277xDBL187___10_27_391_114_Aprx16x2012_B_15B.oka

Replicate 1:

PM11C: J:\Service\Customer\300\348\data\assay\9379\pstu_SIDx6277xDBL187___11_27_379_125_Aprx11x2012_A_20A.oka

Replicate 2:

PM11C: J:\Service\Customer\300\348\data\assay\9391\pstu_SIDx6277xDBL187___11_27_391_114_Aprx16x2012_B_16A.oka

Replicate 1:

PM12B: J:\Service\Customer\300\348\data\assay\9379\pstu_SIDx6277xDBL187___12_27_379_125_Aprx11x2012_A_20B.oka

Replicate 2:

PM12B: J:\Service\Customer\300\348\data\assay\9391\pstu_SIDx6277xDBL187___12_27_391_114_Aprx16x2012_B_16B.oka

Replicate 1:

PM13B: J:\Service\Customer\300\348\data\assay\9379\pstu_SIDx6277xDBL187___13_27_379_125_Aprx11x2012_A_21A.oka

Replicate 2:

PM13B: J:\Service\Customer\300\348\data\assay\9391\pstu_SIDx6277xDBL187___13_27_391_114_Aprx16x2012_B_17A.oka

Replicate 1:

PM14A: J:\Service\Customer\300\348\data\assay\9379\pstu_SIDx6277xDBL187___14_27_379_125_Aprx11x2012_A_21B.oka

Replicate 2:

PM14A: J:\Service\Customer\300\348\data\assay\9391\pstu_SIDx6277xDBL187___14_27_391_114_Aprx16x2012_B_17B.oka

Replicate 1:

PM15B: J:\Service\Customer\300\348\data\assay\9379\pstu_SIDx6277xDBL187___15_27_379_125_Aprx11x2012_A_22A.oka

Replicate 2:

PM15B: J:\Service\Customer\300\348\data\assay\9391\pstu_SIDx6277xDBL187___15_27_391_114_Aprx16x2012_B_18A.oka

Replicate 1:

PM16A: J:\Service\Customer\300\348\data\assay\9379\pstu_SIDx6277xDBL187___16_27_379_125_Aprx11x2012_A_22B.oka

Replicate 2:

PM16A: J:\Service\Customer\300\348\data\assay\9391\pstu_SIDx6277xDBL187___16_27_391_114_Aprx16x2012_B_18B.oka

Replicate 1:

PM17A: J:\Service\Customer\300\348\data\assay\9379\pstu_SIDx6277xDBL187___17_27_379_125_Aprx11x2012_A_23A.oka

Replicate 2:

PM17A: J:\Service\Customer\300\348\data\assay\9391\pstu_SIDx6277xDBL187___17_27_391_114_Aprx16x2012_B_19A.oka

Replicate 1:

PM18C: J:\Service\Customer\300\348\data\assay\9379\pstu_SIDx6277xDBL187___18_27_379_125_Aprx11x2012_A_23B.oka

Replicate 2:

PM18C: J:\Service\Customer\300\348\data\assay\9391\pstu_SIDx6277xDBL187___18_27_391_114_Aprx16x2012_B_19B.oka

Replicate 1:

PM19 : J:\Service\Customer\300\348\data\assay\9379\pstu_SIDx6277xDBL187___19_27_379_125_Aprx11x2012_A_24A.oka

Replicate 2:

PM19 : J:\Service\Customer\300\348\data\assay\9391\pstu_SIDx6277xDBL187___19_27_391_114_Aprx16x2012_B_20A.oka

Replicate 1:

PM20B: J:\Service\Customer\300\348\data\assay\9379\pstu_SIDx6277xDBL187___20_27_379_125_Aprx11x2012_A_24B.oka

Replicate 2:

PM20B: J:\Service\Customer\300\348\data\assay\9391\pstu_SIDx6277xDBL187___20_27_391_114_Aprx16x2012_B_20B.oka

Reference Data Files:

Replicate 1:

PM01 : J:\Service\Customer\300\348\data\assay\9390\pstu_SIDx6276xDAB282___1_27_390_114_Aprx16x2012_A_01A.oka

Replicate 2:

PM01 : J:\Service\Customer\300\348\data\assay\9378\pstu_SIDx6276xDAB282___1_27_378_125_Aprx11x2012_B_05A.oka

Replicate 1:

PM02A: J:\Service\Customer\300\348\data\assay\9378\pstu_SIDx6276xDAB282___2_27_378_125_Aprx11x2012_B_05B.oka

Replicate 2:

PM02A: J:\Service\Customer\300\348\data\assay\9390\pstu_SIDx6276xDAB282___2_27_390_114_Aprx16x2012_A_01B.oka

Replicate 1:

PM03B: J:\Service\Customer\300\348\data\assay\9390\pstu_SIDx6276xDAB282___3_27_390_114_Aprx16x2012_A_02A.oka

Replicate 2:

PM03B: J:\Service\Customer\300\348\data\assay\9378\pstu_SIDx6276xDAB282___3_27_378_125_Aprx11x2012_B_06A.oka

Replicate 1:

PM04A: J:\Service\Customer\300\348\data\assay\9390\pstu_SIDx6276xDAB282___4_27_390_114_Aprx16x2012_A_02B.oka

Replicate 2:

PM04A: J:\Service\Customer\300\348\data\assay\9378\pstu_SIDx6276xDAB282___4_27_378_125_Aprx11x2012_B_06B.oka

Replicate 1:

PM05 : J:\Service\Customer\300\348\data\assay\9390\pstu_SIDx6276xDAB282___5_27_390_114_Aprx16x2012_A_03A.oka

Replicate 2:

PM05 : J:\Service\Customer\300\348\data\assay\9378\pstu_SIDx6276xDAB282___5_27_378_125_Aprx11x2012_B_07A.oka

Replicate 1:

PM06 : J:\Service\Customer\300\348\data\assay\9390\pstu_SIDx6276xDAB282___6_27_390_114_Aprx16x2012_A_03B.oka

Replicate 2:

PM06 : J:\Service\Customer\300\348\data\assay\9378\pstu_SIDx6276xDAB282___6_27_378_125_Aprx11x2012_B_07B.oka

Replicate 1:

PM07 : J:\Service\Customer\300\348\data\assay\9390\pstu_SIDx6276xDAB282___7_27_390_114_Aprx16x2012_A_04A.oka

Replicate 2:

PM07 : J:\Service\Customer\300\348\data\assay\9378\pstu_SIDx6276xDAB282___7_27_378_125_Aprx11x2012_B_08A.oka

Replicate 1:

PM08 : J:\Service\Customer\300\348\data\assay\9390\pstu_SIDx6276xDAB282___8_27_390_114_Aprx16x2012_A_04B.oka

Replicate 2:

PM08 : J:\Service\Customer\300\348\data\assay\9378\pstu_SIDx6276xDAB282___8_27_378_125_Aprx11x2012_B_08B.oka

Replicate 1:

PM09 : J:\Service\Customer\300\348\data\assay\9390\pstu_SIDx6276xDAB282___9_27_390_114_Aprx16x2012_A_05A.oka

Replicate 2:

PM09 : J:\Service\Customer\300\348\data\assay\9378\pstu_SIDx6276xDAB282___9_27_378_125_Aprx11x2012_B_09A.oka

Replicate 1:

PM10 : J:\Service\Customer\300\348\data\assay\9378\pstu_SIDx6276xDAB282___10_27_378_125_Aprx11x2012_B_09B.oka

Replicate 2:

PM10 : J:\Service\Customer\300\348\data\assay\9390\pstu_SIDx6276xDAB282___10_27_390_114_Aprx16x2012_A_05B.oka

Replicate 1:

PM11C: J:\Service\Customer\300\348\data\assay\9390\pstu_SIDx6276xDAB282___11_27_390_114_Aprx16x2012_A_06A.oka

Replicate 2:

PM11C: J:\Service\Customer\300\348\data\assay\9378\pstu_SIDx6276xDAB282___11_27_378_125_Aprx11x2012_B_10A.oka

Replicate 1:

PM12B: J:\Service\Customer\300\348\data\assay\9378\pstu_SIDx6276xDAB282___12_27_378_125_Aprx11x2012_B_10B.oka

Replicate 2:

PM12B: J:\Service\Customer\300\348\data\assay\9390\pstu_SIDx6276xDAB282___12_27_390_114_Aprx16x2012_A_06B.oka

Replicate 1:

PM13B: J:\Service\Customer\300\348\data\assay\9378\pstu_SIDx6276xDAB282___13_27_378_125_Aprx11x2012_B_11A.oka

Replicate 2:

PM13B: J:\Service\Customer\300\348\data\assay\9390\pstu_SIDx6276xDAB282___13_27_390_114_Aprx16x2012_A_07A.oka

Replicate 1:

PM14A: J:\Service\Customer\300\348\data\assay\9378\pstu_SIDx6276xDAB282___14_27_378_125_Aprx11x2012_B_11B.oka

Replicate 2:

PM14A: J:\Service\Customer\300\348\data\assay\9390\pstu_SIDx6276xDAB282___14_27_390_114_Aprx16x2012_A_07B.oka

Replicate 1:

PM15B: J:\Service\Customer\300\348\data\assay\9378\pstu_SIDx6276xDAB282___15_27_378_125_Aprx11x2012_B_12A.oka

Replicate 2:

PM15B: J:\Service\Customer\300\348\data\assay\9390\pstu_SIDx6276xDAB282___15_27_390_114_Aprx16x2012_A_08A.oka

Replicate 1:

PM16A: J:\Service\Customer\300\348\data\assay\9390\pstu_SIDx6276xDAB282___16_27_390_114_Aprx16x2012_A_08B.oka

Replicate 2:

PM16A: J:\Service\Customer\300\348\data\assay\9378\pstu_SIDx6276xDAB282___16_27_378_125_Aprx11x2012_B_12B.oka

Replicate 1:

PM17A: J:\Service\Customer\300\348\data\assay\9378\pstu_SIDx6276xDAB282___17_27_378_125_Aprx11x2012_B_13A.oka

Replicate 2:

PM17A: J:\Service\Customer\300\348\data\assay\9390\pstu_SIDx6276xDAB282___17_27_390_114_Aprx16x2012_A_09A.oka

Replicate 1:

PM18C: J:\Service\Customer\300\348\data\assay\9378\pstu_SIDx6276xDAB282___18_27_378_125_Aprx11x2012_B_13B.oka

Replicate 2:

PM18C: J:\Service\Customer\300\348\data\assay\9390\pstu_SIDx6276xDAB282___18_27_390_114_Aprx16x2012_A_09B.oka

Replicate 1:

PM19 : J:\Service\Customer\300\348\data\assay\9378\pstu_SIDx6276xDAB282___19_27_378_125_Aprx11x2012_B_14A.oka

Replicate 2:

PM19 : J:\Service\Customer\300\348\data\assay\9390\pstu_SIDx6276xDAB282___19_27_390_114_Aprx16x2012_A_10A.oka

Replicate 1:

PM20B: J:\Service\Customer\300\348\data\assay\9378\pstu_SIDx6276xDAB282___20_27_378_125_Aprx11x2012_B_14B.oka

Replicate 2:

PM20B: J:\Service\Customer\300\348\data\assay\9390\pstu_SIDx6276xDAB282___20_27_390_114_Aprx16x2012_A_10B.oka
